# Decoupling the Effects of the Amyloid Precursor Protein From Amyloid-β Plaques on Axonal Transport Dynamics in the Living Brain

**DOI:** 10.3389/fncel.2019.00501

**Published:** 2019-12-03

**Authors:** Christopher S. Medina, Taylor W. Uselman, Daniel R. Barto, Frances Cháves, Russell E. Jacobs, Elaine L. Bearer

**Affiliations:** ^1^Department of Pathology, University of New Mexico Health Sciences Center, Albuquerque, NM, United States; ^2^Zilkha Neurogenetic Institute, Keck School of Medicine, University of Southern California, Los Angeles, CA, United States; ^3^California Institute of Technology, Pasadena, CA, United States

**Keywords:** transgenic mice for Alzheimer’s disease investigation, amyloid precursor protein (APP), fast microtubule-based axonal transport, CA3 of the hippocampus, dentate gyrus and septal nuclei, manganese-enhanced magnetic resonance imaging (MEMRI), cholinergic neurons, Swedish and Indiana mutation (APP^SwInd^)

## Abstract

Amyloid precursor protein (APP) is the precursor to Aβ plaques. The cytoplasmic domain of APP mediates attachment of vesicles to molecular motors for axonal transport. In APP-KO mice, transport of Mn^2+^ is decreased. In old transgenic mice expressing mutated human (APP^SwInd^) linked to Familial Alzheimer’s Disease, with both expression of APP^SwInd^ and plaques, the rate and destination of Mn^2+^ axonal transport is altered, as detected by time-lapse manganese-enhanced magnetic resonance imaging (MEMRI) of the brain in living mice. To determine the relative contribution of expression of APP^SwInd^ versus plaque on transport dynamics, we developed a Tet-off system to decouple expression of APP^SwInd^ from plaque, and then studied hippocampal to forebrain transport by MEMRI. Three groups of mice were compared to wild-type (WT): Mice with plaque and APP^SwInd^ expression; mice with plaque but suppression of APP^SwInd^ expression; and mice with APP^SwInd^ suppressed from mating until 2 weeks before imaging with no plaque. MR images were captured before at successive time points after stereotactic injection of Mn^2+^ (3–5 nL) into CA3 of the hippocampus. Mice were returned to their home cage between imaging sessions so that transport would occur in the awake freely moving animal. Images of multiple mice from the three groups (suppressed or expressed) together with C57/B6J WT were aligned and processed with our automated computational pipeline, and voxel-wise statistical parametric mapping (SPM) performed. At the conclusion of MR imaging, brains were harvested for biochemistry or histopathology. Paired *T*-tests within-group between time points (*p* = 0.01 *FDR corrected*) support the impression that both plaque alone and APP^SwInd^ expression alone alter transport rates and destination of Mn^2+^ accumulation. Expression of APP^SwInd^ in the absence of plaque or detectable Aβ also resulted in transport defects as well as pathology of hippocampus and medial septum, suggesting two sources of pathology occur in familial Alzheimer’s disease, from toxic mutant protein as well as plaque. Alternatively mice with plaque without APP^SwInd^ expression resemble the human condition of sporadic Alzheimer’s, and had better transport. Thus, these mice with APP^SwInd^ expression suppressed after plaque formation will be most useful in preclinical trials.

## Introduction

Alzheimer’s disease (AD), a progressive, debilitating illness, ends in death. No cure or even any effective treatment is yet available. Senile plaques composed of amyloid beta fibrils together with dystrophic neurites, and neurofibrillary tangles of phosphorylated tau protein diagnose the disease. The discoveries that amyloid precursor protein (APP), the parent of amyloid beta, attaches microtubule motors to vesicular cargo for axonal transport (Kamal et al., 2000; Satpute-Krishnan et al., 2006; Seamster et al., 2012), and that tau protein binds microtubules and also plays roles in transport, have led to proposals that transport defects accompany AD, either in causative or synergistic ways (Stokin and Goldstein, 2006; Bearer, 2012). Projections from the hippocampus to septal and forebrain structures are involved in cognition, and a site of neurodegeneration in AD (Sviatko and Hangya, 2017).

We use manganese-enhanced magnetic resonance imaging (MEMRI) to study axonal transport in the living brain (Bearer et al., 2007a, b, 2009a,b, 2018; Zhang et al., 2010; Gallagher et al., 2012, 2013). Manganese, a divalent metal cation, is a calcium indicator for MRI that enters activated neurons through voltage-gated calcium channels, secondarily enters organelles, and is transported via microtubule motors within axons. MEMRI traces neuronal projections from sites of stereotaxic injections and thereby maps their connections (Pautler et al., 1998, 2003; Inoue et al., 2011). Manganese, a paramagnetic ion, gives a strong hyper-intense signal by T_1_-weighted MR (Koretsky and Silva, 2004; Silva et al., 2004). Mn^2+^ is delivered by precisely localized stereotaxic injection and the expected location of the injection site confirmed by 0.5 h post-injection MR image, after which the mouse is returned to its home cage. Transport then occurs in the awake freely moving mouse and retrospectively imaged at successive time points. After each imaging session, the mouse is returned to its home cage (Bearer et al., 2007b, 2009a, 2018; Gallagher et al., 2012; Medina et al., 2017a). Distal accumulation of Mn^2+^, giving hyper-intense T_1_-weighted signal, can be identified in MRI images using statistical parametric mapping (Friston, 1996), revealing the anatomy of projections and rates of transport. Using this technique, we recently reported that in aging mice expressing a FAD-associated mutant APP, APP^SwInd^, and dense plaques, transport is significantly decreased and terminal destinations are altered (Bearer et al., 2018).

Here, we investigate whether plaques decrease transport in the absence of mutant APP expression, or whether mutant APP on its own, in the absence of plaque, can affect transport within the living brain. A double transgenic mouse carrying the APP^SwInd^ gene under control of the Tet-off system allows separation of plaque from APP^SwInd^ expression (Jankowsky et al., 2005; Bearer et al., 2018). To measure transport in the living brain, we performed stereotaxic injections of Mn^2+^ into CA 3 of the hippocampus and imaged cohorts of living mice before and at 0.5, 6, and 24 h afterward. The relative large size of the hippocampal to basal forebrain fiber bundles renders them ideal for imaging the progression of the Mn^2+^–induced hyper-intense signal at 100^3^ μm resolution with reasonable (∼40 min) scan times.

## Materials and Methods

### Animals

To decouple the effect of mutant APP expression on transport from the effect of amyloid-β plaque, we used transgenic mice that overexpress a mutant human APP gene with expression controlled by the Tet-off promoter (Jankowsky et al., 2005). The human APP695 gene carrying both the Swedish and Indiana mutations, each associated with Familial Alzheimer’s Disease (FAD), was used in this study. We will refer to this gene as “APP^SwInd^.” Mice with the APP^SwInd^ mutation were deposited at The Jackson Laboratory (JAX) (Bar Harbor, ME, United States) by the Borchelt lab, B6.Cg-Tg(TetO-APPSwInd, strain 34845), and were only available to us from JAX as frozen embryos. We recovered breeding pairs and back-crossed them to C57/B6J (also referred to as JAX strain 000664) to obtain a monogenic APP^SwInd^ stock congenic with C57/B6J (Bearer et al., 2018).

Monogenic tTA mice [JAX 007004B6.Cg-Tg (CamIIatTA) 1Mmay-DboJ], a transgenic line that expresses the TetO transcription activator (tTA) in a C57/B6J background was bred with the monogenic APP^SwInd^ mice using IACUC-approved breeding protocols at UNM to produce double transgenic, TetO/APP^SwInd^ mice. Tail snip genotyping was done by PCR at Transnetyx to monitor the genotypes of all mice at each generation. Four genotypes of offspring were produced with this breeding protocol: no transgene, one or the other transgene (monogenic tTA or monogenic APP^SwInd^) and double transgenic mice (both tTA and APP^SwInd^). We generated 38 double transgenic mice, both male and female, aged to 5–6 months for our experiments. No significant difference was found between sexes. We also analyzed data from 12 mice with a C57/B6J background (JAX strain 000664) for a wild-type (WT) group aged to 4–6 months acquired previously which we use as normal controls (Gallagher et al., 2012). This is part of a multi-year project which includes generating the monogenic APP^SwInd^ line, back = crossing for 10 generations in the C57/B6J strain, mating with tTA and aging the progeny. We began by developing our imaging protocol, performing power analysis on pilot WT C57/B6J mice, and comparing to other readily available mutants that did not require aging (Gallagher et al., 2012; Medina et al., 2017a; Bearer et al., 2018). We previously reported no effect of monogenic APP^SwInd^ or tTA on axonal transport of Mn^2+^, hippocampal dentate width or density and numbers of cholinergic neurons in the medial septal nuclei even at older ages (13–15 months) (Bearer et al., 2018). Hence we did not study these monogenics in this investigation but rather used congenic wild type as normal controls.

### Ethics Statement

All protocols involving animals in this study were approved by Institutional Animal Care and Use Committees (IACUC) of the California Institute of Technology and of the University of New Mexico. Number of animals was based on our statistical power analyses.

### Tet-Off Experimental Design

**FIGURE 1:**
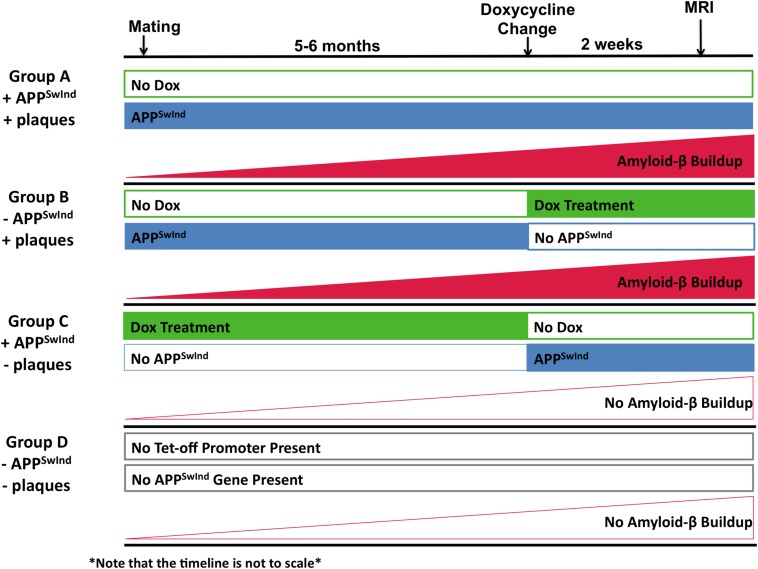
Diagram of experimental design to decouple APP^SwInd^ expression from plaque burden. Expression of the APP^SwInd^ gene is controlled by a doxycycline-sensitive Tet-off promoter. Treatment with doxycycline (Dox) (green boxes), while expression of APP^SwInd^ (blue boxes). Group A (+APP^SwInd^, +Amyloid-β/plaques) (*n* = 11) was never treated with doxycycline. Thus Group A mice expressed APP^SwInd^ throughout their lives and produced amyloid-β plaques (red rising plane indicating the gradual rise in plaque accumulation). Group B (– APP^SwInd^, +Amyloid-β/plaques) (*n* = 8) was begun on doxycycline 2 weeks before imaging. This allowed for plaque formation from the APP^SwInd^ that was expressed throughout the life of the mice, but no APP^SwInd^ would be expressed during the time of experiment. Group C (+APP^SwInd^, –Amyloid-β/plaques) (*n* = 13) female mice were treated with doxycycline prior to mating and off spring continued on doxycylcine until 2 weeks before imaging. APP^SwInd^ was therefore expressed during the experiment but amyloid-β plaques would not be present due to the limited amount of time in which sufficient plaque-forming APP^SwInd^ would be expressed. Group D (–APP^SwInd^, –Amyloid-β/plaques) (*n* = 12) did not carry either the Tet-off promoter construct or the Tet-off promoter-driven APP^SwInd^ insert (Indicated by gray outline boxes).

Double transgenic mice were divided into three groups, which had expression of APP^SwInd^ switched on or off at different time points of the life of the mice (Figure 1 and Table 1). This allowed us to create mice that had either APP^SwInd^ expression or amyloid-β plaque accumulation, but not both, in two of our experimental groups. We were thus able to observe the specific effects of each of these two factors for the first time.

**TABLE 1:** Mice.

**Cohort**	**Age**	**Strain/numbers of animals**
Group A (+APP^SwInd^, +Amyloid-β/plaques)	5–6 months	C57/B6J, APPSwInd/tTA (*n* = 11)
Group B (−APP^SwInd^, +Amyloid-β/plaques)	5–6 months	C57/B6J, APPSwInd/tTA (*n* = 8)
Group C (+APP^SwInd^, −Amyloid-β/plaques)	5–6 months	C57/B6J, APPSwInd/tTA (*n* = 13)
WT Group (−APP^SwInd^, −Amyloid-β/plaques)	4–6 months	C57/B6J (*n* = 12)

*Additional animals for each cohort were used for histology (see section Materials and Methods), and some animals expired during the time course of experiments.*

**FIGURE 2:**
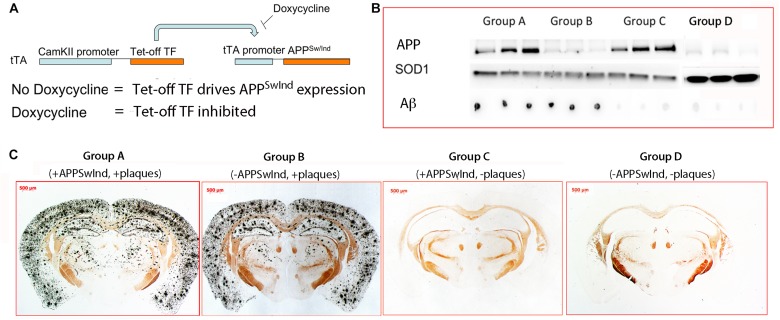
Diagram of the transgene and confirmation of doxycycline suppression. **(A)** In order to toggle the expression of APP^SwInd^, its expression was driven by a doxycycline-sensitive Tet-off transcription factor, tTA, carried on a separate transgene under control of the neuron-specific CAMKII promoter (Mayford et al., 1996). Whenever mice were not under doxycycline treatment, APP^SwInd^ is expressed in neurons expressing tTA. Doxycycline treatment of double transgenic mice carrying both transgenes inhibits the Tet-off transcription factor from binding to the Tet-Off promoter preventing it from driving APP^SwInd^ expression. **(B)** Western and dot blots of mouse brain extract from three individual mice in each group of APP^SwInd^ mice, and Group D, WT. Note that Group A and B are positive for Aβ, and Group A and C express APP^SwInd^ (hAPP). SOD1 serves as a loading control that confirms amount of extract loaded in all lanes. APP^SwInd^ (hAPP) in Group D appears similar as Group B, suggesting the hAPP band is cross-reaction with mouse APP. **(C)** Examples of histologic sections from each of the four Groups, A, B, C, and D, stained for plaque. As expected, Group A and B display classic silver-stained plaques throughout the cortex, hippocampus and some in deeper structures, such as the thalamus, caudate-putamen and lateral geniculate. Previously we reported a few plaques in the septal regions of 6 months old APP^SwInd^ mice (Bearer et al., 2018). In contrast, Group C and D, as expected, have no detectible plaque.

Using the Tet-off promoter, APP^SwInd^ is normally expressed but silenced with doxycycline (Figure 2A). Our first group, “Group A” (+APP^SwInd^ + Amyloid-β/plaques) (*n* = 11), was never treated with doxycycline and thus expressed APP^SwInd^ throughout the 5–6 months of the experiment. Because of the continuous expression of APP, these mice were expected to accumulate amyloid-β and plaques (Bearer et al., 2018). Our second group, “Group B” (−APP^SwInd^ + Amyloid-β/plaques) (*n* = 8), was started on doxycycline 2 weeks before the MR images were captured and kept on doxycycline until sacrifice, 9–15 days, and histology and biochemistry studies performed. For doxycycline, we employed Bio-Serv “Dox Diet” (200 mg/kg doxycycline, Frenchtown, NJ, United States) to shut down APP^SwInd^ expression. Previous studies have shown that 2 weeks on this diet is sufficient to decrease APP^SwInd^ levels by >95% (Jankowsky et al., 2005). The expected dose at 200 mg/kg of chow for each animal was 1 mg dox per day. Chow was replenished 1–2 times per week. These mice were expected to have plaques but no APP^SwInd^ expression.

Our third group, “Group C” (+APP^SwInd^ – Amyloid-β/plaques) (*n* = 13), was fed the doxycycline diet via the mother before mating (i.e., before conception) reported to reduce APP^SwInd^ expression by 98% (Jankowsky et al., 2005), and then continued on doxycycline after birth until 2 weeks before MR imaging. These mice expressed APP^SwInd^ at the time of experiment, but would not be expected to have amyloid-β plaque, as APP^SwInd^ was not expressed long enough for plaque formation (Jankowsky et al., 2005), although a small amount of various C-terminal fragments may be produced since the mutations increase production of proteolytic products of APP, including Amyloid-β (Johnston et al., 1994). The WT Group D (− APP^SwInd^ – Amyloid-β/plaques) (*n* = 12) did not carry either Tet-off promoter or the APP^SwInd^ gene and did not have a buildup of amyloid-β plaque. We previously demonstrated that the monogenic mice, either APP^SwInd^ or tTA, were not statistically difference from wild type even at old ages (Bearer et al., 2018).

### Stereotaxic Injection

First, we collected pre-injection MR images to serve as a baseline for alignments. Then, stereotaxic injections were performed as described in previous publications (Bearer et al., 2007b, 2018; Gallagher et al., 2012; Medina et al., 2017a). Briefly, mice were anesthetized and secured in place with a stereotaxic frame while 3–5 nL of 200 mM aqueous Mn^2+^ was injected into the *cornu ammonis* field 3 (CA3) of the right hippocampus (target coordinates *x* = 3.2 mm right of midline, *y* = −4.1 mm posterior to bregma, *z* = 3.4 mm down from surface of brain). A quartz micropipette guided by computer-assisted stereotaxic injector (myNeuroLAB.com, IL, United States) was used to perform the injection over a 5 min period. Mn^2+^ was used as the tract tracer for MR imaging proposes. We choose 3–5 nL of 200 mM because it is an amount that we previously demonstrated to be sufficient to “saturate” the uptake system, but small enough to be non-toxic (Gallagher et al., 2012). The volume of injectate was kept small to enable precise placement.

The expected injection sites were confirmed by measurements in single images taken 0.5 h after injection after alignments. The coordinates of the center of the hypo-intense region, with the highest concentration of Mn^2+^, was determined using MRIcron (Rorden et al., 2007)^1^ and recorded in relation to bregma as defined by Paxinos and Franklin atlas (Paxinos and Franklin, 2001) and plotted onto our 3D mouse brain template atlas as previously described (Medina et al., 2017a).

### Time-Lapse MR Imaging of Axonal Transport

**FIGURE 3:**
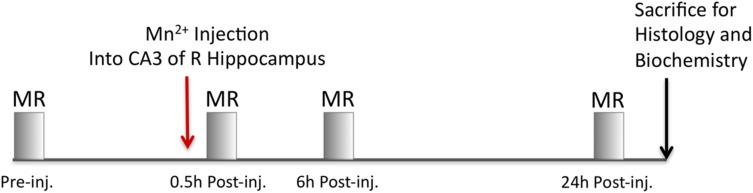
Imaging timeline. A pre-injection image is first captured to serve as a non-enhanced image for alignments and background subtraction of MR images for each respective mouse. Next, 3–5 nL of Mn^2+^ is injected into CA3 of the hippocampus and the mouse immediately imaged by MR. After imaging, the mice are recovered from anesthesia and returned to their home cage. MR images are captured from the same mice at 6 and 24 h after injection. Between each imaging session, the mice are removed from the scanner, recovered from anesthesia and allowed to freely move about in their home cage. At the conclusion of the imaging sessions, mice are sacrificed.

Mice were imaged using an 11.7 T 89 mm vertical bore Bruker BioSpin Avance DRX500 scanner (Bruker BioSpin Inc., Billerica, MA, United States) equipped with a Micro2.5 gradient system equipped with a 35 mm linear birdcage radio frequency (RF) coil. For each mouse, a pre-injection image was captured and then images were captured at 0.5, 6, and 24 h after injection (Figure 3). During imaging, the mice were kept under anesthesia with 1–1.5% isoflurane. The head of each mouse was secured in a Teflon stereotaxic unit within the RF coil to aid in reproducible placement within the column and to minimize movement. Respiration and temperature were monitored continuously during data acquisition and maintained within normal ranges. Our imaging parameters are similar to previous MEMRI studies carried out by this group (Gallagher et al., 2012; Medina et al., 2017a; Bearer et al., 2018). We employed a 3D multi-echo RARE imaging sequence that is both T_1_- and T_2_-weighted, with RARE factor of 4 and these parameters: 4 averages, TR/TE eff = 250 ms/12 ms; matrix size of 160 × 128 × 88; FOV 16 mm × 12.8 mm × 8.8 mm; yielding 100 μm isotropic voxels with a 46 min scan duration. We identified the “scan time” as the midpoint of the scan. No significant difference in timing within or between groups was found. The Mn^2+^ intensity is captured by T_1_-weighting and anatomical detail increased by T_2_-weighting. No Mn^2+^ leakage into ventricular or vascular systems was apparent either by visual inspection or even after statistical comparisons within group between time points, which confirmed no additional intensity in the ventricles over time. For time-line of the pre-injection image, the injection and post-injection time-lapse imaging, please refer to Figure 3.

### Histology and Immunochemistry

Histology contributed to three aspects of this study: (1) To confirm the presence or absence of plaques between Groups; (2) To determine the histopathology of the hippocampus; and (3) To analyze the cholinergic neurons in a target destination. The mice were sacrificed after MR imaging, fixed and submitted for histologic analysis. Histologic sections of the WT Group from other studies performed by this lab were also included (Gallagher et al., 2012; Bearer et al., 2018). Subsets of these sections were selected for microscopic analysis and examination as specified in figure legends.

Mice were either sacrificed by cardiac perfusion of 30 ml of warm heparinized phosphate buffer saline (PBS), followed by 30 ml of room temperature 4% paraformaldehyde (PFA) in PBS as reported previously (Bearer et al., 2007b, 2018), or by cervical dislocation followed by rapid brain harvest for biochemistry (Bearer et al., 2018). Perfused mice were decapitated and then the head rocked in 4% PFA and PBS overnight at 4°C and stored in PBS-azide. Each brain was released from the skull prior to processing and fixed again for 1–3 more days. The brains were then sent to NeuroScience Associates (Knoxville, TN, United States) for gelatin embedding and serial sectioning coronally in register at 35 μm thickness. Up to 25 brains are usually embedded together in a single block. Sections are collected in 24 sequential cups such that each cup contained serial sections across the whole brain at approximately 840 μm intervals. For histochemistry and immunochemistry, alternate cups containing serial sections of 20 brains from Groups A, B, C, and D (5 of each) were stained in a variety of ways: Campbell-Switzer stain (Schonheit et al., 2004), a silver stain for plaques and tangles; thionine (Nissl) for microscopic anatomy; and immunohistochemistry for cholinergic neurons stained with anti-ChAT antibodies (Bearer et al., 2007b, 2018) (NeuroScience Associates, Knoxville, TN, United States).

A Zeiss V8 stereoscope was used to perform whole brain microscopy of sections, equipped with an AxioCam running AxioVision 4.8. For higher magnification, images were captured on a Nikon Eclipse Ci brightfield clinical microscope with a DS L3 cooled digital camera. Adobe Photoshop CS 5.1 (Adobe Systems Incorporated, San Jose, CA, United States) was used to crop and prepare images for publication. The C57/B6J atlas was used as reference for the position of images relative to bregma^2^ (Paxinos and Franklin, 2001).

### Western Blots and Dot Blots

Brains were harvested within 90 s of decapitation and plunged into liquid N_2_. Briefly, 10 mg of brain was first homogenized in ice-cold Tissue-Protein Extraction Reagent (T-PER, 78510; Pierce, now Thermo Fisher Scientific, Waltham, MA, United States) with protease (P8340; Sigma-Aldrich) and phosphatase (P5726; Sigma-Aldrich) inhibitor cocktails with a Dounce homogenizer for 1 min, and then sonicated in a Bronson sonifier set at 20% for 20 s on ice as described (Bhaskar et al., 2010; Bearer et al., 2018). After microfuging at 14,000 rpm in an Eppendorf microfuge for 15 min, the supernatant and pellet are separated and stored at −80°C. For SDS protein gels, 20–50 μg of protein-containing extract (supernatant) was boiled in Laemmle gel sample buffer and loaded onto a 10% SDS-PAGE for electrophoretic separation (Thermo Fisher Scientific, Waltham, MA, United States) (Seamster et al., 2012). Proteins were transferred to nitrocellulose in a Hoeffer tank transfer device overnight in transfer buffer. For dot blots, 3 μl of brain extract was dotted onto nitrocellulose and allowed to dry for 20 min, washed in blocking buffer for 20 min, and then probed with primary anti-Aβ monoclonal antibody MOAB (#MABN254, Millipore, Burlington, MA, United States) at 1:1000; or for SOD1 with polyclonal anti-SOD1, 1:1000 (#AB5482, Millipore, Burlington, MA, United States) (Bearer et al., 2018). HRP-conjugated secondary antibodies were goat anti-mouse (#AP308P, Millipore, Burlington, MA, United States) and goat anti-rabbit (#AP307P, Millipore, Burlington, MA, United States) diluted at 1:1000 in blocking buffer. Western blots were probed with the SOD1 antibodies as a loading control, and 6E10 anti-APP monoclonal antibody #SIG-39320 at 1:1000 (Covance, Brea, CA, United States). To compare mouse and human APP expression, Western blots were stained in parallel with monoclonal 22C10, or with a polyclonal rabbit antibody raised against the conserved C-terminus of APP with pan-species reactivity at 1:1000 (#MAB348. Millipore, Burlington, MA, United States), and 6E10, a monoclonal against the non-conserved N-terminus of human APP, which is non-reactive with mouse APP, at 1:1000 (Covance, Brea, CA, United States). Stained blots were imaged with ECL Clarity (BioRad #170-5060, Hercules, CA, United States) on BioRad ChemiDoc XR+ using the Magic Mark ladder (Invitrogen #LC5602, Carlsbad, CA, United States) and Kaleidoscope ladder (BioRad #161-0375, Hercules, CA, United States). Intensity of bands was calculated using ImageJ^3^.

### MR Image Analysis

In order to prepare the MR images for unbiased voxel-wise statistical analysis, several previously published processing steps must be performed on the images (Delora et al., 2016; Medina et al., 2017b). This processing pipeline includes extracting the brain from non-brain structures in the MR images, a process we name “skull-stripping” (Delora et al., 2016) followed by normalizing the intensity scale of the images and finally aligning the images (Medina et al., 2017b). Briefly, 3D MR images were converted to 16-bit images in the NIfTI (.nii) file format (Neuroimaging Informatics Technology Initiative). FSL (FMRIB software library) (Analysis Group, Oxford, United Kingdom)^4^ (Smith et al., 2004; Jenkinson et al., 2012) was used to check for uniformity of header file geometry. Voxel size was uniformly assigned using the 3drefit program from AFNI (Analysis of Functional NeuroImages)^5^ (Cox, 1996; AFNI: software for analysis). A script developed by the Jacobs lab while at California Institute of Technology (Currently at University of Southern California) was used to correct for B1 inhomogeneity in MIPAV (Medical Image Processing, Analysis and Visualization) (McAuliffe et al., 2001)^6^. The mode of the intensity histogram was scaled to be equal across all images using a custom MATLAB (MathWorks, Natick, MA, United States) script developed by the Jacobs lab. Both of these scripts are posted as Supplementary Material by Medina et al. (2017b). We then generate a template mask to mask out non-brain material using Brain Surface Extractor within BrainSuite11 followed by manual refinement, also in BrainSuite11 (Shattuck and Leahy, 2001). The rest of the images are skull-stripped in our automated skull-stripper program using this template mask as a reference image for the skull-stripper algorithm (Delora et al., 2016). After skull-stripping, the images were then corrected again for B-field inhomogeneity and then the grayscale normalized again to correct for any influence non-brain material may have had during the first normalization. The scaled images were registered to our mouse brain template (MacKenzie-Graham et al., 2004; Medina et al., 2017b) using our skull-stripper program. The high resolution template living mouse brain image what captured at 24 h post-infusion of Mn^2+^ systemically at 80 μm^3^ voxel resolution for 4.5 h T_1_ scan time. This template image can be obtained from the *Current Protocols in Molecular Biology* website for our publication (Medina et al., 2017b) which describes in more detail how our mouse brain template was created. After alignments, including both rigid body and non-linear procedures, the images are smoothed with full-width half-maximum (FWHM) Gaussian kernel set to 0.3 mm using the SPM software package (Statistical Parametric Mapping) (UCL, London, United Kingdom)^7^ (Friston, 1996; Ashburner and Friston, 2001).

To create a map of Mn^2+^ enhancement at successive time points as a marker of axonal transport of the Mn^2+^ from the injection site, we performed SPM analysis^8^ following similar methods to those we previously reported (Bearer et al., 2007b, 2018; Gallagher et al., 2012; Medina et al., 2017a). Within-group between-time-point analyses were compared using pairwise *t*-tests (6 h > 0.5 h and 24 h > 0.5 h) with a *p*-value of 0.01 with a false discovery rate (FDR) correction for multiple comparisons (repeated measures) to obtain an unbiased, whole brain, voxel-wise map of transport dynamics in SPM in all four cohorts.

### Region of Interest Analysis

Volumes of 0.5 mm^3^ (5 × 5 × 5 voxels) were primarily selected as regions of interest based on SPM signal observed throughout cohorts and secondarily adjusted according to brain anatomy. Using FSL (Analysis Group FMRIB, Oxford, United Kingdom), selected regions were propagated across fully aligned images specified by image coordinates, and mean intensity within each region was calculated for each animal, at every time point. Mean intensities for 30 m post injection images were set to 1 and the same adjustment carried through to respective animal’s mean intensity at 6 and 24 h post injection. Statistical analyses of ROIs were performed in GraphPad Prism using a two-way, repeated measures ANOVA, with Bonferroni’s *post hoc* test for multiple comparisons (GraphPad Software Inc, La Jolla, CA, United States). Statistical outcomes were compiled into a table and line graphs were created to display mean intensity ± standard error of the mean over time within group. Main effect of time, condition, and interaction were also analyzed (see Supplementary Materials). Statistical significance between groups at each time point within ROIs is indicated on the graphs by asterisks. A 3D template image and ROIs were superimposed in FSLeyes to create orthogonal slice images intersecting at each ROI’s central voxel. ROI locations were determined by each ROI’s center voxel in relation to bregma as described above.

### Measurements of the Dentate Gyrus and Stereology of ChAT Neurons of the Medial Septal Nuclei

For the dentate gyrus, we measured the width of the granule cell layer with the measurement tool in Fiji (Rueden et al., 2017). We could not count cells as nuclei overlapped in these 35 μm sections. Widths were estimated using Nissl-stained sections in the anterior-dorsal region of the hippocampus in 2–3 sections of five animals for each group. Both right and left hippocampi for each animal were measured at 300 μm intervals along both arms of the dentate, resulting in 6–9 measurements per hippocampus.

Stereology of the cholinergic neurons in the medial septal nuclei was performed as previously described (Bearer et al., 2018). Sections through the septal region were stained for choline-acetyl transferase (ChAT) by immunohistochemistry as described above. We segmented the medial septal nuclei (MSN) in histologic sections (3 sections per mouse) based on classical landmarks (Allen Mouse Brain Reference Atlas) (Lein et al., 2007) and Paxinos and Franklin (Paxinos and Franklin, 2001) (also see Supplementary Material in Bearer et al., 2018). ChAT-positive cells were counted using the Stereo Investigator software (Microbrightfield Stero Investigator Software, Williston, VT, United States) for design-based stereology. Area within the contour was calculated by the stereology software. The fractionator probe (Microbrightfield Stero Investigator Software, Williston, VT, United States) was applied to count all ChAT-stained cells in the MSN within a given contour, with the anterior commissure as the landmark. Cells were only included if the entire cell body was inside the contour. Both the number of cholinergic neurons counted and the density of cells were averaged for each group. The density of ChAT-sained cells was based on the average number of neurons counted divided by the average area occupied by the counted cells. Results were grouped into 4 cohorts according to genotype and condition, and analyzed with GraphPad Prism using a one-way ANOVA, and Tukey’s *post hoc* tests. All stereological protocols were performed on an Olympus DSU inverted microscope at the University of New Mexico Fluorescence Microscopy Shared Resource Center.

## Results

### Separation of Plaque Formation From APP^SwInd^ Expression

We examined five mice from each of our four groups of tTA/APP^SwInd^ double transgenic, Group A, Group B, Group C, and Group D for expression of the APP^SwInd^ transgene by Western blot, the presence of Aβ by dot blot, and presence of plaques by histochemistry (Figures 2B,C). As expected, Groups A and B displayed numerous plaques, while Groups C and D had no detectable plaques (Figure 2C). This correlated with dot blots for Aβ (Figure 2B), which were also positive for Groups A and B and not C and D. In contrast, also as expected by our experimental design (Figure 1), the APP^SwInd^ protein was detected by Western blot in Group A and C but not Group B and D (Figure 2B). Note that all lanes of Groups A, B and C were equivalently loaded, as shown by the SOD1 loading control, while more extract was loaded in Group D. Despite this higher amount, little signal is detected for APP or Aβ. The low signal from the anti-APP antibody in Group B and D likely represents mouse APP, which is also expressed in these suppressible mutants, although some signal in Group B may be the reported low amount (5%) of APP^SwInd^ transgene expression (Jankowsky et al., 2005).

These results confirm that Group A has both plaque and APP^SwInd^ expression, that Group B has plaques and little to no APP^SwInd^ expression, and that Group C has no plaques but APP^SwInd^ expression at the same level as Group A, the non-suppressed control. We measured the intensity of bands in Western blots probed with a pan-specific APP antibody (MAb 22C10), which recognizes both mouse and human APP, and found that levels of transgene overexpression are approximately 3.2-fold that of mouse APP in Group A and C, as we previously reported (Bearer et al., 2018).

### Placement of the Injection Site Was Similar for All Individual Mice in the Study

**FIGURE 4:**
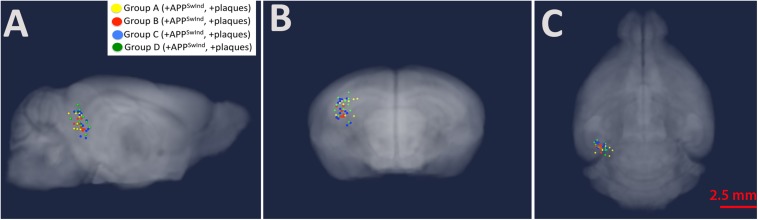
Injection sites mapped onto a gray-scale MR image shown in transparent three-dimensions. Group A, yellow; Group B, red; Group C, blue; Group D (WT), green **(A)** sagittal; **(B)** coronal; and **(C)** axial. Note the tight clustering of all injections sites in CA3 of the right hippocampus. Also see Table 2.

Using MR images to locate the hypo-intense injection site 0.5 h after injection allows us to confirm accurate placement and to compare the position of injection across all mice (Figure 4 and Table 2). The distance from the centroid of all injections along any particular plane was 0.97 mm or less and the overall distance from the centroid was 1.02 mm or less. Analysis with and without the two furthest outliers did not make a difference to the SPM maps: the mouse that deviated 0.97 mm deep (z-plane) from the centroid, and another that had the largest overall distance from the centroid at 1.02 mm. Variance of all injection sites in each of the three dimensions was 0.08 mm lateral to midline (x-plane), 0.05 mm in the anterior-posterior direction (y-plane) and 0.17 deep (z-plane).

**TABLE 2:** Actual injection site locations measured for the 30 m post-injection MRI.

**Cohort**	**Injection sites measured in MR image 0.5 h post-injection (mm)**		
	**X (L-R)**	**Y (front-back)**	**Z (deep)**
Group A (+APP^SwInd^, +Amyloid-β/plaques)	2.73 ± 0.37	−4.81 ± 0.21	2.42 ± 0.42
Group B (−APP^SwInd^, +Amyloid-β/plaques)	3.03 ± 0.17	−4.62 ± 0.19	2.80 ± 0.26
Group C (+APP^SwInd^, −Amyloid-β/plaques)	2.96 ± 0.25	−4.55 ± 0.23	2.62 ± 0.54
WT Group (−APP^SwInd^, −Amyloid-β/plaques)	2.90 ± 0.32	−4.63 ± 0.30	2.43 ± 0.45
Farthest Distance from Centroid (mm)	0.81	0.65	0.97

### Gray-Scale Normalization and Image Alignments Are Comparable Across All Four Cohorts

**FIGURE 5:**
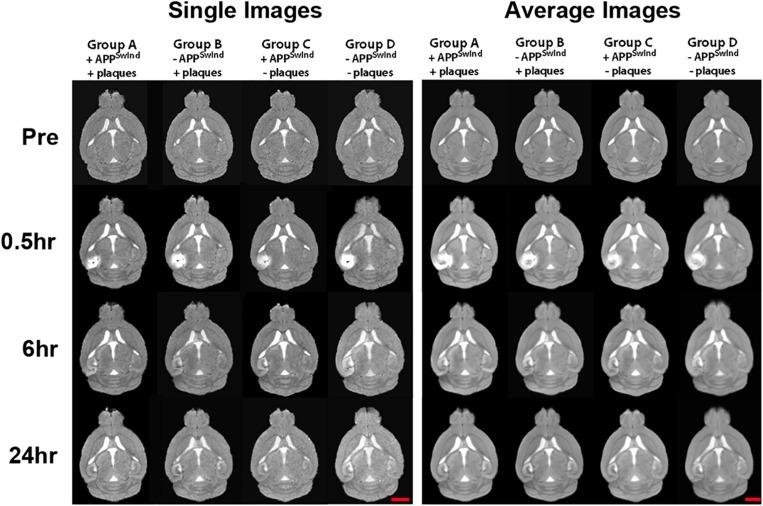
Examples of single images and averaged images for each cohort at each time point as indicated. Note consistency between individual mice before alignments (Sample Images) and similarities between average images (Average Images) for each time point within each of the four cohorts. Gray-scale normalization renders all images in the same intensity scale and anatomical alignments have improved anatomical detail. Mag bar = 2.5 mm.

Our skull stripping, normalization and alignment process renders all images from these experiments co-equal in terms of intensity and anatomy (Figure 5). As shown, individual normalized aligned images are nearly indistinguishable from the averaged image for that cohort at that time point. Since our analysis is based on the gray-scale intensity and the anatomical position, these processing steps are critical for interpretation. Our normalization process aligns the mode of the intensity histogram according to our custom MATLAB script (Medina et al., 2017b). We validate these steps by visual inspection as well as by quantitative comparisons of the intensity histograms before and after normalization, and the three dimensional matrix of the averaged and individual images.

### Statistical Parametric Maps of Mn^2+^ Progress From Hippocampus to Forebrain

**FIGURE 6:**
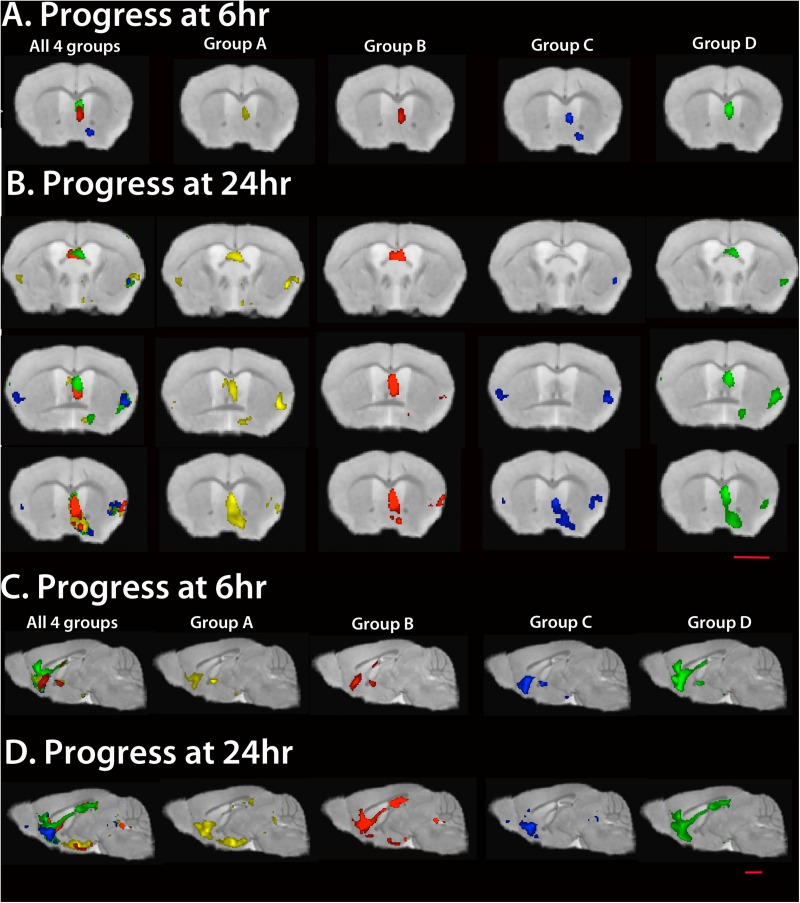
Statistical maps of each cohort. Maps are projected onto our high resolution live mouse template image (Medina et al., 2017b). All SPMs are shown at FDR *p* < 0.01. To correlate anatomy of statistically significant voxels, we project all four Groups onto the same image (left column of images, **A–D**). **(A)** Coronal slices at 6 h post Mn^2+^ are shown through the anterior commissure and corpus callosum for all four and for each Group separately. **(B)** Coronal slices at 24 h post Mn^2+^ injection through fornix (top), medial septum (middle) and basal forebrain (bottom). **(C)** Sagittal slices through the midline show progress at 6 h. Note that all three transgenic groups display less anterior-most signal in the pre-orbital forebrain than WT. **(D)** Sagittal slices at 24 h show that only Group B catches up to WT, with plaques and suppression of APP^SwInd^ expression for 2 weeks prior to imaging. Of note is the lag in Group A, with both plaques and APP^SwInd^ expression, and in Group C, with no plaques and APP^SwInd^ expression. Mag bars = 2.5 mm. Please see Supplementary Table S1 for *T*-values for each cohort at each time point; see Supplementary Video S1 for 3D rendering of these maps.

To compare the anatomical position of statistically significant voxels, we projected statistical maps of all four cohorts, as well as from each cohort, onto the same gray scale image in three dimensions at each time point (Figure 6 and Supplementary Video S1). Coronal and sagittal slices from these 3D images show progress of the Mn^2+^-induced intensity increase from the injection site in the hippocampus along the fimbria and fornix into the basal forebrain, known projections from CA3. The location of the Mn^2+^ intensity signal depends on uptake at the injection site in the hippocampus (Bearer et al., 2007b); axonal transport by the anterograde kinesin-driven microtubule-based system (Bearer et al., 2007a; Medina et al., 2017a), and possibly the dynein-driven retrograde transport system (Matsuda et al., 2010); APP expression (Gallagher et al., 2012) and levels of other cargo-motor receptors; and accumulation of Mn^2+^ along the way and at its distal destinations (Bearer et al., 2018). To avoid uptake rates as possible confounders, we inject an amount of Mn^2+^ that saturates uptake mechanisms (Medina et al., 2017a). However there is the caveat that plaque in CA3 of the hippocampus may affect uptake capacity in Groups A and B.

Visual comparisons of the four groups provide three main observations: (1) WT (green) displays the most distal signal at 6 h in both coronal and sagittal slices. Hence either APP^SwInd^ expression or plaques or both limit normal uptake/transport/accumulation of Mn^2+^; (2) Only Group B (red) with plaques but not overexpression catches up to WT at 24 h while Group C (blue) displays limited distal accumulation and does not appear to progress from 6 to 24 h; (3) Both Groups with APP^SwInd^ expression (Groups A and C) display delayed transport, and both groups with plaques (Groups A and B) display proximal (septal region) accumulations. We consider delayed transport as indicated by how far from the injection site the accumulation appears.

To discover a basis for these differences, we performed three additional analyses. Since our statistical maps are binary, and only show whether voxels are significant above a certain threshold, we performed region of interest (ROI) intensity measurements along the projections across all four cohorts and three time points to determine the actual average degree of intensity between cohorts. To determine if alterations in the hippocampus could interfere with uptake, we analyzed the morphology in histologic sections of the hippocampus. And to determine if there were cellular changes in the destination, the septum, we performed quantification of septal areas, numbers of cholinergic cells and their density by stereology of a subset of mice in all four cohorts.

### Region of Interest (ROI) Analysis Shows That Plaque Alters Axonal Transport, and That APP^SwInd^ Expression Compromises Axonal Transport More Than Plaque

Group B represents the condition of sporadic Alzheimer’s disease, which is not replicated in any other mouse model, as all others constitutively express mutated proteins. Group B, in contrast, models the condition of plaque in the absence of mutant protein expression, as is found in human sporadic Alzheimer’s disease. Thus for the first time, we report the effect of plaque alone on axonal transport on the important memory circuit from hippocampus to basal forebrain.

**FIGURE 7:**
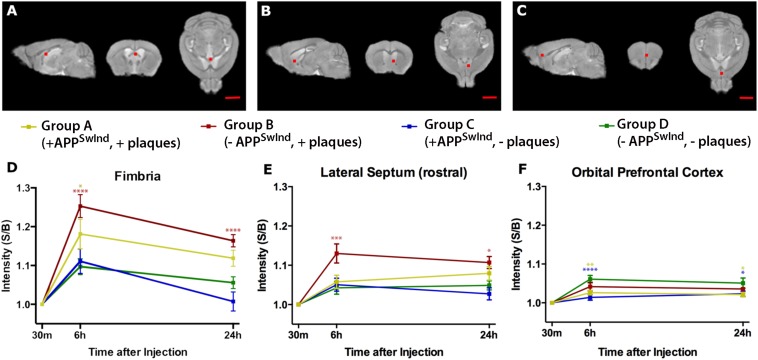
Region of interest analysis shows that expression of APP^SwInd^ delays arrival of Mn^2+^- increased intensity at distal destinations, and presence of plaque increases Mn^2+^ accumulation at proximal locations. Panels **(A–C)** shows the location on a template image in orthogonal slices of the measured 5 × 5 × 5 voxel cube (0.5 mm^3^) correlating with the graphs below each set of images. In **(A)**, coordinates in the middle of the cube are: *x* = –0.2, *y* = 0.5, *z* = –2.4 mm; in **(B),** *x* = –0.7, *y* = –0.6, *z* = –3.6 mm; in **(C)**, *x* = –0.3, *y* = –1.9, *z* = –2.6 mm, where *x* is medial-lateral from midline; y is anterior-posterior from bregma; *z* is dorsal-ventral from bregma. **(D–F)** Graphs show intensity measures at each time point for each Group at each location as a percent change in mean intensity over time relative to the 30 m measurement: Group A, +APP^SwInd^, +plaques (yellow); Group B, –APP^SwInd^, +plaques (red), Group C, +APP^SwInd^, –plaques (blue), and Group D, –APP^SwInd^, –plaques (green). Error bars represent standard error of the mean. Statistical significance is indicated by asterisks from a two-way ANOVA with a repeated measures correction (Bonferroni) for multiple comparisons between groups, with only comparisons to WT shown (^****^P < 0.0001; ^∗∗∗^*P* < 0.001; ^∗∗^*P* < 0.01; ^∗^*P* < 0.05). Non-significant results have no asterisks. GraphPad Prism software was used to prepare graphs and perform two-way ANOVA with repeated measures with Bonferroni correction for multiple comparisons. Mag bars = 2.5 mm. See Supplementary Table S2 for flexible factorial analysis of effects of time, condition, and interactions as well as significance of comparisons between other groups not shown here.

Intensity increased in both fimbria and lateral septum at 6 h most dramatically in Group B, with plaques but with suppression of APP^SwInd^ expression for 2 weeks before imaging (Figure 7). This increase was statistically significant compared to all other Groups, and especially significant when compared to WT (*p* < 0.0001 for fimbria and <0.001 for lateral septum, Bonferroni corrected) (see Supplementary Table S2). Thus, plaque alone affects the dynamics of transport.

Group A, with plaques and APP^SwInd^ expression, similar to other mouse models of AD, had less accumulation at 6 h in fimbria than Group B, but this intensity was still significantly different form WT (*p* < 0.05, Bonferroni). Group A caught up to WT at 24 h, as the difference was no longer statistically significant. At 24 h, Group B sustains its statistically significant increased signal in the fimbria (*p* < 0.05, Bonferroni), and in the lateral septum (*p* < 0.05, Bonferroni). At 6 or 24 h in the lateral septum, all three other cohorts are not significantly different from WT. In contrast, at more distal locations, e.g., the orbital prefrontal cortex, both Groups expressing APP^SwInd^, Group A with plaque and Group C without, display less intensity suggesting delayed axonal transport at both time points. At 6 h, Group A, *p* < 0.01; Group C, with no plaque, *p* < 0.001, Bonferroni. At 24 h these two Groups, A and C, continue to display less intensity than WT (Group A, *p* < 0.05; Group C, *p* < 0.05). In contrast, Group B with plaques but no APP^SwInd^ expression is not significantly different from WT at either time point in the orbital prefrontal cortex.

### Lifelong Expression of APP^SwInd^ Affects the Dentate Gyrus of the Hippocampus

**FIGURE 8:**
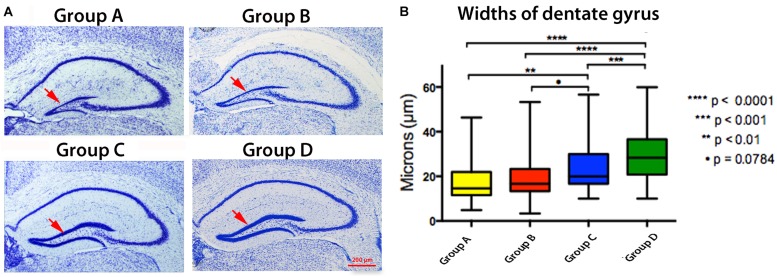
Effect of Plaque and APP^SwInd^ on hippocampal histology. **(A)** Examples of thionine (Nissl) stained sections from each group as indicated. **(B)** Widths of the hippocampal dentate gyrus with statistical comparisons between groups shown as quartile box plots. Means, indicated by the horizontal black line inside the box, are: Group A, 16.7 ± 7.2 μm; Group B, 19.3 ± 9 μm; Group C, 23.5 ± 9.6 μm; Group D, 28.1 ± 12μm. Note that Group A is significantly different from C and D. Group B is less significantly different to Group C than A suggesting it may have partially recovered from the effects of APP^SwInd^ expression, even though this group has plaques comparable to those in Group A, or that Group A continued to lose dentate width in the short time that APP^SwInd^ was shut-off in Group B. Also note in Group C that short-term expression of APP^SwInd^ may have affected the dentate, or that there was a low APP^SwInd^ expression during the doxycycline treatment. The diminished dentate gyrus width is unlikely to be due to tTA expression since no such effect was observed in tTA monogenics in the C57/B6J strain (Han et al., 2012; Bearer et al., 2018).

To determine whether altered transport is due to anatomical changes in the hippocampus secondary to the timing of APP^SwInd^ expression or the presence of plaques, we examined the hippocampus of the same mice imaged by MR in histologic sections stained with thionine (Nissl) (Bearer et al., 2018) (Figure 8). Although diminished dentate gyrus width in tTA Tet-off mice in some strain backgrounds has been reported (Han et al., 2012), our mice, back-crossed into the same C57/B6J background of our APP^SwInd^ strain, and do not display this effect (Bearer et al., 2018). Old APP^SwInd^ expressing mice (13–15 months of age) had thinner dentate gyri obvious in microscopic images and measurable by stereology, while Tet-off monogenic mice had normal dentate gyral thickness (Bearer et al., 2018) (Figure 8A). Group A is a younger version of the old mice we previously reported–and at 6 months. These mice also have decreased dentate gyrus readily visible in histologic sections (Figure 8A) that was measurably different (Group A, 16.7 ± 7.2 μm versus Group D, 28.1 ± 12 μm, *p* < 0.0001) (Figure 8B). Group B mice, with lifelong APP^SwInd^ only suppressed shortly before sacrifice, displayed dentate gyrus loss but not quite as great as Group A (19.3 ± 9 μm versus 16.7 ± 7.2 μm) although this difference is not statistically significant. This suggests that the dentate damage may be recoverable if plaque formation is turned off even if plaques remain. Group C, with expression suppressed throughout life until just before imaging, also had some dentate loss (23.5 ± 9.6 μm versus Group D, 28.1 ± 12 μm *p* < 0.001), but less than Groups A and B.

These data show that plaque alters transport independent of mutant protein expression, but to a lesser extent than when together with mutant proteins. We further studied phospho-tau deposition (Supplementary Figure S1). Clusters of mouse p-tau aggregates were found in Groups A and B but not C, in a distribution resembling plaques shown in Figure 2. This suggests that p-tau aggregation is not involved in delayed transport in Group C. Furthermore, suppression of mutant protein may allow hippocampal recovery even if suppression does not result in plaque resorption (Jankowsky et al., 2005). The data also suggest that while even a short time and/or low expression of APP^SwInd^ does not produce plaque (see Figure 2) as in Group C, it yet causes hippocampal changes. These results support our interpretation of the MEMRI transport data, where in Group B transport is more robust and dentate gyrus may be recovering after suppression of the transgene despite pre-existing plaques. Alternatively, expression of APP^SwInd^ alone, without plaque, is deleterious, since expression without plaques in Group C alters transport, and may damage the dentate gyrus.

### Effect of APP^SwInd^ Expression Versus Plaque on Cholinergic Neurons in the Septum

**FIGURE 9:**
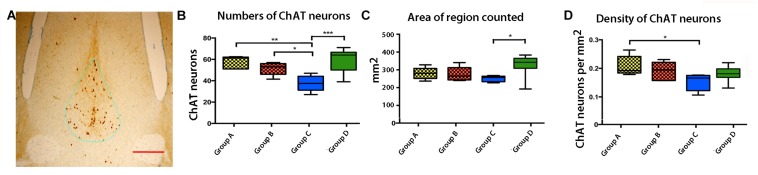
Effects of APP^SwInd^ expression and plaque on basal forebrain cholinergic neurons. **(A)** Example of the medial septal region containing cholinergic neurons, stained for ChAT (brown) and outlined in the stereology set-up used for measurement (blue line). **(B–D**) Box and whisker plots showing quartile distributions of the number of ChAT positive cells, the area in the outlined septal region, and the density of cells (counts/area) in each of the four groups, as labeled. ^∗^*p* < 0.01; ^∗∗^*p* < 0.001, ^∗∗∗^*p* < 0.0001 as indicated.

Cholinergic neuron health depends on retrograde transport of NGF (Tuszynski et al., 2005, 2015; Salehi et al., 2006) and also may depend on anterograde transport from the presynaptic neurons in the hippocampus. Cholinergic neurons of the septum are known to become atrophic and degenerate in Alzheimer’s disease (Sviatko and Hangya, 2017). Since these neurons are the targets for CA3 projections, decreased Mn^2+^ transport from CA3 could be secondary to fewer target neurons. To determine if APP^SwInd^ expression and/or plaque affected the population of septal cholinergic neurons, we stained all four of our groups by immuno-histochemistry for choline acetyl transferase (ChAT), a marker of cholinergic neurons (Figure 9). We determined the number, the area and the density of ChAT neurons in each group by stereology. The most significant decrease of ChAT positive cells was found in Group C (Figure 9B, *p* < 0.0001 from WT). Group C had significantly less ChAT positive cells than either of the other APP^SwInd^ groups. Both the area of the septal region measured and the density of ChAT neurons were diminished in Group C compared to WT (*p* < 0.001 and *p* < 0.01 respectively). Thus, loss of ChAT neurons could be either a consequence of short term decreased transport from hippocampus to this region, or conversely decreased ChAT targets could be affecting transport in Group C. Since Group C has no plaque, this effect on cholinergic neurons must be a consequence of APP^SwInd^ protein expression or its secondary effects, possibly decoupled from a direct effect of transport. Such secondary effects would include the sudden increased production of toxic APP C-terminal fragments upon removal of the doxycycline, lack of growth factor signaling, or paucity of synaptic building blocks normally delivered by transport to the presynaptic nerve terminal.

## Discussion

Here we show by live, longitudinal MRI of the whole brain, differences in axonal transport dynamics with and without plaque in mouse models of Alzheimer’s disease. The unique mouse model created for these studies can be manipulated to have either a high plaque burden with no mutated APP expression, or no plaques and expression of mutated APP. By shutting down mutant APP expression after plaques have formed, we dissociate APP expression from plaque burden, and thereby test the effect of each independently on transport. In all other currently available mouse lines carrying mutant APP, expression is constitutive and plaques develop. Hence no other system has been available to decouple plaques from expression, which is the condition of sporadic Alzheimer’s disease in humans. By time-lapse imaging of the whole brain in living animals, we show for the first time the dynamics of axonal transport in the central nervous system, and how FAD proteins impact this physiologic process. Furthermore, by quantitative examination of hippocampal morphology and cell counts in the basal forebrain cholinergic system, we begin to dissect the impact of transport dynamics on neuronal health. A next step will be to learn how these transport dynamics affect cognition.

Our results support previous ideas that mutated APP is toxic, independent of plaque (Gunawardena and Goldstein, 2001, 2004; Rodrigues et al., 2012; Weissmiller et al., 2015; Xu et al., 2016). We show in live imaging of the whole brain that expression of APP^SwInd^ in the absence of plaque decreases axonal transport from hippocampus to basal forebrain, and affects the number of cholinergic neurons in the septum. While plaque also has an effect on transport success, this effect it is much less than the expression of mutated APP, and has been invisible in mouse models with constitutive mutant protein expression.

Why might this be? Our mutated APP contains two mutations that increase gamma secretase activity and are dominant mutations in human families, the Swedish double mutation (Thordardottir and Graff, 2018) and the Indiana single amino acid substitution (Murrell et al., 1991). Hence inheritance of one copy of this gene results in 100% penetrant Alzheimer’s disease–the mutation poisons endogenous APP. Our mice have normal mouse APP and a 3.2-fold expression of this poisonous mutated protein. Our results predict that expression of mutated APP, independent of plaque, diminishes nourishing axonal transport to critical distal targets–septal region of the basal forebrain– and may have other, non-transport-related toxic effects. This diminished transport together with toxic APP^SwInd^ is deleterious for cholinergic neurons, and likely other distal target cells not witnessed here.

This does not mean that aggregation of Aβ into plaque has no consequences. Plaque also caused pathology, although less dramatic in assays of axonal transport, and cholinergic neuron number. Plaque alone did not result in decreased transport in these mice. But plaque decreased the width–i.e., the number of cells–in the dentate gyrus, indicating either cell death or lack of neuronal renewal, which occurs through stem cell division in that region of the mouse brain and likely in human as well.

How might we be wrong in our interpretation of these imaging and histology results? Differences in the injection site placement were minimal, and thus unlikely to account for the differences in transport observed between groups. Perhaps the mutated protein changes Mn^2+^ uptake through neuronal calcium channels. However, APP FAD mutations do not affect calcium signaling (Stieren et al., 2010). Hence this seems a less likely reason. Increased expression of APP itself, even without a mutation, might reduce axonal transport and damage cholinergic neurons. This explanation is also unlikely, since in our peer-reviewed publication on Down Syndrome, also performed with MEMRI, we found increased transport with 1.5-fold expression of APP, a gene on the triplicated chromosome, and decreased numbers of cholinergic neurons in the basal forebrain (Bearer et al., 2007b). Plaque may also affect uptake in the hippocampus, although the data from Group B, with better transport than A despite hippocampal plaques, argues against that possibility. Furthermore, decreased transport in Group C, with no plaque, suggests that plaque-related uptake differences are not a major factor in our results.

Others have also reported that this APP mutation causes transport defects in cell culture systems, where transport is imaged as particle movements rather than intensity changes (Rodrigues et al., 2012). Transport defects may occur through cleavage of the cytoplasmic domain of APP by gamma secretase, which removes the kinesin-binding domain that mediates cargo motility (Kamal et al., 2000, 2001; Satpute-Krishnan et al., 2006). Such cleavage would also produce a soluble cytoplasmic domain that would bind kinesin and inhibit it from associating with other organelles. Hence not only would the APP-containing vesicles lose transport capability, but other organelles would have to compete with the cytoplasmic fragment to acquire motors. We have shown that soluble peptides derived from the cytoplasmic domain of APP when injected into the squid axon inhibit transport (Satpute-Krishnan et al., 2006; Bearer and Wu, 2019) through binding to kinesin (Seamster et al., 2012). Primary hippocampal cultures or slices from the mice used in this study could be implemented to tease apart the consequences of APP^SwInd^ expression and plaque on transport of specific cargoes.

Since our Down Syndrome findings were with a small (1.5-fold) increased expression of normal APP, which in mouse does not produce plaques, the possibility remains that increased protein concentration due to the 3.2-fold excess expression in our APP^SwInd^ transgenic mouse may non-specifically inhibit transport. Indeed, many disorders of cognition involve protein aggregates that likely represent an abnormally high level of protein in the cytoplasm. How cognition depends on transport will be an important area of future study. It will be interesting to know if other cognitive disorders share a transport defect, since the biological basis of cognition remains a mystery. What is the role of axonal transport in this critical human mental process?

Currently it is unclear whether transport deficits cause pathology or vice versa, and ever present question in the field of axonal transport research. Here we aimed to address this but found that in our 6-month old mice, even those with suppressed APP^SwInd^ expression, there was both transport defects and pathology, with decreased ChAT neurons and dentate width. Future experiments in younger mice may identify an age in which the hippocampus and ChAT neurons are not yet impacted in the disease models, but the transport phenotype is present. Such a finding would provide stronger evidence for the importance of transport deficits to the (possibly) subsequent pathology. Alternatively, lifelong expression of APP^SwInd^ may have deleterious effects that we have not studied here. Alternatively, if, as results suggest, suppressing APP^SwInd^ expression results in some recovery despite the continued presence of plaque, future experiments with a longer period of suppression could be valuable.

Finally, we may consider how this new information might lead to therapeutic interventions. If suppression of Amyloid-β production results in dentate and ChAT neuronal recovery as our results with Group B suggest, then targeting peptide production rather than plaques may have significant therapeutic potential. Since the dentate represents a region of active neurogenesis, the impact of toxic APP fragments may interfere with this process in addition to promoting death of mature neurons. Recent developments using vaccine-based approaches to both tauopathies (Maphis et al., 2019) and APP-related disorders (Chackerian et al., 2006; Chackerian, 2010; Li et al., 2010) may provide vehicles for such new treatments aimed at suppressing toxic proteins. We might also ask if transport can be restored, and if so, does cognition improve?

## Data Availability Statement

The datasets generated for this study are available on request to the corresponding author.

## Ethics Statement

The animal study was reviewed and approved by the Institutional Animal Care and Use Committees (IACUC) of the California Institute of Technology and of the University of New Mexico.

## Author Contributions

EB designed the experiments, performed the microscopy and biochemistry, and co-wrote the manuscript. CM did the analysis and drafted the manuscript. TU performed the SPM and ROI analysis, and edited the manuscript. DB helped with the 3D rendering and guided TU in the use of Amira. FC performed the stereology and statistical analysis. RJ performed the MR imaging and edited the manuscript.

## Conflict of Interest

The authors declare that the research was conducted in the absence of any commercial or financial relationships that could be construed as a potential conflict of interest.

## Footnotes

1 http://people.cas.sc.edu/rorden/mricron/index.html

2 http://www.mbl.org/atlas170/atlas170_frame.html

3 https://imagej.nih.gov/ij/

4 https://fsl.fmrib.ox.ac.uk/fsl/fslwiki/

5 https://afni.nimh.nih.gov/

6 https://mipav.cit.nih.gov

7 http://www.fil.ion.ucl.ac.uk/spm/doc/

8 http://www.fil.ion.ucl.ac.uk/spm/doc/biblio/

## References

[B1] Ashburner J. Friston K. J. (2001). Why voxel-based morphometry should be used. *Neuroimage* 14 1238–1243. 10.1006/nimg.2001.0961 11707080

[B2] Bearer E. L. (2012). HSV, axonal transport and Alzheimer’s disease: in vitro and in vivo evidence for causal relationships. *Future Virol.* 7 885–899. 10.2217/fvl.12.81 23335944 PMC3546524

[B3] Bearer E. L. Falzone T. L. Zhang X. Biris O. Rasin A. Jacobs R. E. (2007a). Role of neuronal activity and kinesin on tract tracing by manganese-enhanced MRI (MEMRI). *Neuroimage* 37(Suppl. 1), S37–S46. 17600729 10.1016/j.neuroimage.2007.04.053 PMC2096707

[B4] Bearer E. L. Zhang X. Jacobs R. E. (2007b). Live imaging of neuronal connections by magnetic resonance: robust transport in the hippocampal-septal memory circuit in a mouse model of Down syndrome. *Neuroimage* 37 230–242. 10.1016/j.neuroimage.2007.05.010 17566763 PMC2074885

[B5] Bearer E. L. Manifold-Wheeler B. C. Medina C. S. Gonzales A. G. Chaves F. L. Jacobs R. E. (2018). Alterations of functional circuitry in aging brain and the impact of mutated APP expression. *Neurobiol. Aging* 70 276–290. 10.1016/j.neurobiolaging.2018.06.018 30055413 PMC6159914

[B6] Bearer E. L. Wu C. (2019). Herpes simplex virus, Alzheimer’s disease and a possible role for Rab GTPases. *Front. Cell Dev. Biol.* 7:134. 10.3389/fcell.2019.00134 31448273 PMC6692634

[B7] Bearer E. L. Zhang X. Biris O. Jacobs R. E. (2009a). “Imaging biophysics of axonal transport with MEMRI: optic tract transport is altered in mouse model of Alzheimer’s disease,” in *Proceedings of the International Society for Magnetic Resonance in Medicine. Scientific Meeting and Exhibition*, (Concord, CA: International Society for Magnetic Resonance in Medicine). PMC4509602 26207099

[B8] Bearer E. L. Zhang X. Janvelyan D. Boulat B. Jacobs R. E. (2009b). Reward circuitry is perturbed in the absence of the serotonin transporter. *Neuroimage* 46 1091–1104. 10.1016/j.neuroimage.2009.03.026 19306930 PMC2693299

[B9] Bhaskar K. Konerth M. Kokiko-Cochran O. N. Cardona A. Ransohoff R. M. Lamb B. T. (2010). Regulation of tau pathology by the microglial fractalkine receptor. *Neuron* 68 19–31. 10.1016/j.neuron.2010.08.023 20920788 PMC2950825

[B10] Chackerian B. (2010). Virus-like particle based vaccines for Alzheimer disease. *Hum Vaccin.* 6 926–930. 10.4161/hv.7.1.12655 20864801

[B11] Chackerian B. Rangel M. Hunter Z. Peabody D. S. (2006). Virus and virus-like particle-based immunogens for Alzheimer’s disease induce antibody responses against amyloid-beta without concomitant T cell responses. *Vaccine* 24 6321–6331. 10.1016/j.vaccine.2006.05.059 16806604

[B12] Cox R. W. (1996). AFNI: software for analysis and visualization of functional magnetic resonance neuroimages. *Comput. Biomed. Res. Int. J.* 29 162–173. 10.1006/cbmr.1996.0014 8812068

[B13] Delora A. Gonzales A. Medina C. S. Mitchell A. Mohed A. F. Jacobs R. E. (2016). A simple rapid process for semi-automated brain extraction from magnetic resonance images of the whole mouse head. *J. Neurosci. Methods* 257 185–193. 10.1016/j.jneumeth.2015.09.031 26455644 PMC4910826

[B14] Friston K. J. (1996). “Statistical parametric mapping and other analysis of functional imaging data,” in *Brain Mapping: The Methods*, eds Toga A. W. Mazziotta J. C. (San Diego, CA: Academic Press), 471 10.1016/b978-012372560-8/50036-x

[B15] Gallagher J. J. Zhang X. Hall F. S. Uhl G. R. Bearer E. L. Jacobs R. E. (2013). Altered reward circuitry in the norepinephrine transporter knockout mouse. *PLoS One* 8:e57597. 10.1371/journal.pone.0057597 23469209 PMC3587643

[B16] Gallagher J. J. Zhang X. Ziomek G. J. Jacobs R. E. Bearer E. L. (2012). Deficits in axonal transport in hippocampal-based circuitry and the visual pathway in APP knock-out animals witnessed by manganese enhanced MRI. *Neuroimage* 60 1856–1866. 10.1016/j.neuroimage.2012.01.132 22500926 PMC3328142

[B17] Gunawardena S. Goldstein L. S. (2001). Disruption of axonal transport and neuronal viability by amyloid precursor protein mutations in Drosophila. *Neuron* 32 389–401. 10.1016/s0896-6273(01)00496-2 11709151

[B18] Gunawardena S. Goldstein L. S. (2004). Cargo-carrying motor vehicles on the neuronal highway: transport pathways and neurodegenerative disease. *J. Neurobiol.* 58 258–271. 10.1002/neu.10319 14704957

[B19] Han H. J. Allen C. C. Buchovecky C. M. Yetman M. J. Born H. A. Marin M. A. (2012). Strain background influences neurotoxicity and behavioral abnormalities in mice expressing the tetracycline transactivator. *J. Neurosci.* 32 10574–10586. 10.1523/JNEUROSCI.0893-12.2012 22855807 PMC3431916

[B20] Inoue T. Majid T. Pautler R. G. (2011). Manganese enhanced MRI (MEMRI): neurophysiological applications. *Rev. Neurosci.* 22 675–694. 10.1515/RNS.2011.048 22098448 PMC3269402

[B21] Jankowsky J. L. Slunt H. H. Gonzales V. Savonenko A. V. Wen J. C. Jenkins N. A. (2005). Persistent amyloidosis following suppression of Abeta production in a transgenic model of Alzheimer disease. *PLoS Med.* 2:e355. 10.1371/journal.pmed.0020355 16279840 PMC1283364

[B22] Jenkinson M. Beckmann C. F. Behrens T. E. Woolrich M. W. Smith S. M. (2012). FSL. *Neuroimage* 62 782–790. 10.1016/j.neuroimage.2011.09.015 21979382

[B23] Johnston J. A. Cowburn R. F. Norgren S. Wiehager B. Venizelos N. Winblad B. (1994). Increased beta-amyloid release and levels of amyloid precursor protein (APP) in fibroblast cell lines from family members with the Swedish Alzheimer’s disease APP670/671 mutation. *FEBS Lett.* 354 274–278. 10.1016/0014-5793(94)01137-0 7957938

[B24] Kamal A. Almenar-Queralt A. LeBlanc J. F. Roberts E. A. Goldstein L. S. (2001). Kinesin-mediated axonal transport of a membrane compartment containing beta-secretase and presenilin-1 requires APP. *Nature* 414 643–648. 10.1038/414643a 11740561

[B25] Kamal A. Stokin G. B. Yang Z. Xia C. H. Goldstein L. S. (2000). Axonal transport of amyloid precursor protein is mediated by direct binding to the kinesin light chain subunit of kinesin-I. *Neuron* 28 449–459. 10.1016/s0896-6273(00)00124-0 11144355

[B26] Koretsky A. P. Silva A. C. (2004). Manganese-enhanced magnetic resonance imaging (MEMRI). *NMR Biomed.* 17 527–531. 10.1002/nbm.940 15617051

[B27] Lein E. S. Hawrylycz M. J. Ao N. Ayres M. Bensinger A. Bernard A. (2007). Genome-wide atlas of gene expression in the adult mouse brain. *Nature* 445 168–176. 17151600 10.1038/nature05453

[B28] Li Q. Y. Gordon M. N. Chackerian B. Alamed J. Ugen K. E. Morgan D. (2010). Virus-like peptide vaccines against Abeta N-terminal or C-terminal domains reduce amyloid deposition in APP transgenic mice without addition of adjuvant. *J. Neuroimmun. Pharmacol.* 5 133–142. 10.1007/s11481-009-9183-1 20066498 PMC13019653

[B29] MacKenzie-Graham A. Lee E. F. Dinov I. D. Bota M. Shattuck D. W. Ruffins S. (2004). A multimodal, multidimensional atlas of the C57BL/6J mouse brain. *J. Anat.* 204 93–102. 10.1111/j.1469-7580.2004.00264.x 15032916 PMC1571243

[B30] Maphis N. M. Peabody J. Crossey E. Jiang S. Jamaleddin Ahmad F. A. Alvarez M. (2019). Qss Virus-like particle-based vaccine induces robust immunity and protects against tauopathy. *NPJ Vaccin.* 4:26. 10.1038/s41541-019-0118-4 31231552 PMC6547647

[B31] Matsuda K. Wang H. X. Suo C. McCombe D. Horne M. K. Morrison W. A. (2010). Retrograde axonal tracing using manganese enhanced magnetic resonance imaging. *NeuroImage* 50 366–374. 10.1016/j.neuroimage.2010.01.008 20074651

[B32] Mayford M. Bach M. E. Huang Y. Y. Wang L. Hawkins R. D. Kandel E. R. (1996). Control of memory formation through regulated expression of a CaMKII transgene. *Science* 274 1678–1683. 10.1126/science.274.5293.1678 8939850

[B33] McAuliffe M. J. Lalonde F. M. McGarry D. Gandler W. Csaky K. Trus B. L. (2001). “Medical image processing, analysis and visualization in clinical research,” in *Proceedings of the IEEE COMPUTER-BASED MEDICAL SYSTEMS (CBMS’01)*, (Piscataway, NJ: IEEE), 381–386.

[B34] Medina C. S. Biris O. Falzone T. L. Zhang X. Zimmerman A. J. Bearer E. L. (2017a). Hippocampal to basal forebrain transport of Mn2+ is impaired by deletion of KLC1, a subunit of the conventional kinesin microtubule-based motor. *Neuroimage* 145 44–57. 10.1016/j.neuroimage.2016.09.035 27751944 PMC5457905

[B35] Medina C. S. Manifold-Wheeler B. Gonzales A. Bearer E. L. (2017b). Automated computational processing of 3-D mr images of mouse brain for phenotyping of living animals. *Curr. Protoc. Mol. Biol.* 119 21A–29A. 10.1002/cpmb.40 28678440 PMC6195809

[B36] Murrell J. Farlow M. Ghetti B. Benson M. D. (1991). A mutation in the amyloid precursor protein associated with hereditary Alzheimer’s disease. *Science* 254 97–99. 10.1126/science.1925564 1925564

[B37] Pautler R. G. Mongeau R. Jacobs R. E. (2003). In vivo trans-synaptic tract tracing from the murine striatum and amygdala utilizing manganese enhanced MRI (MEMRI). *Magn. Reson. Med.* 50 33–39. 10.1002/mrm.10498 12815676

[B38] Pautler R. G. Silva A. C. Koretsky A. P. (1998). In vivo neuronal tract tracing using manganese-enhanced magnetic resonance imaging. *Magn. Reson. Med*. 40 740–748. 10.1002/mrm.1910400515 9797158

[B39] Paxinos G. Franklin K. (2001). *The Mouse Brain in Stereotaxic Coordinates.* San Diego, CA: Academic Press, 296.

[B40] Rodrigues E. M. Weissmiller A. M. Goldstein L. S. (2012). Enhanced beta-secretase processing alters APP axonal transport and leads to axonal defects. *Hum. Mol. Genet.* 21 4587–4601. 10.1093/hmg/dds297 22843498 PMC3471392

[B41] Rorden C. Karnath H. O. Bonilha L. (2007). Improving lesion-symptom mapping. *J. Cogn. Neurosci.* 19 1081–1088. 10.1162/jocn.2007.19.7.1081 17583985

[B42] Rueden C. T. Schindelin J. Hiner M. C. DeZonia B. E. Walter A. E. Arena E. T. (2017). ImageJ2: imageJ for the next generation of scientific image data. *BMC Bioinformatics* 18:529. 10.1186/s12859-017-1934-z 29187165 PMC5708080

[B43] Salehi A. Delcroix J. D. Belichenko P. V. Zhan K. Wu C. Valletta J. S. (2006). Increased App expression in a mouse model of Down’s syndrome disrupts NGF transport and causes cholinergic neuron degeneration. *Neuron* 51 29–42. 10.1016/j.neuron.2006.05.022 16815330

[B44] Satpute-Krishnan P. DeGiorgis J. A. Conley M. P. Jang M. Bearer E. L. (2006). A peptide zipcode sufficient for anterograde transport within amyloid precursor protein. *Proc. Natl. Acad. Sci. U.S.A.* 103 16532–16537. 10.1073/pnas.0607527103 17062754 PMC1621108

[B45] Schonheit B. Zarski R. Ohm T. G. (2004). Spatial and temporal relationships between plaques and tangles in Alzheimer-pathology. *Neurobiol. Aging* 25 697–711. 10.1016/s0197-4580(04)00079-x 15165691

[B46] Seamster P. E. Loewenberg M. Pascal J. Chauviere A. Gonzales A. Cristini V. (2012). Quantitative measurements and modeling of cargo-motor interactions during fast transport in the living axon. *Phys. Biol.* 9:055005. 10.1088/1478-3975/9/5/055005 23011729 PMC3625656

[B47] Shattuck D. W. Leahy R. M. (2001). Automated graph-based analysis and correction of cortical volume topology. *IEEE Trans. Med. Imaging* 20 1167–1177. 10.1109/42.963819 11700742

[B48] Silva A. C. Lee J. H. Aoki I. Koretsky A. P. (2004). Manganese-enhanced magnetic resonance imaging (MEMRI): methodological and practical considerations. *NMR Biomed.* 17 532–543. 10.1002/nbm.945 15617052

[B49] Smith S. M. Jenkinson M. Woolrich M. W. Beckmann C. F. Behrens T. E. Johansen-Berg H. (2004). Advances in functional and structural MR image analysis and implementation as FSL. *Neuroimage* 23(Suppl. 1), S208–S219. 15501092 10.1016/j.neuroimage.2004.07.051

[B50] Stieren E. Werchan W. P. El Ayadi A. Li F. Boehning D. (2010). FAD mutations in amyloid precursor protein do not directly perturb intracellular calcium homeostasis. *PLoS One* 5:e11992. 10.1371/journal.pone.0011992 20700539 PMC2916833

[B51] Stokin G. B. Goldstein L. S. (2006). Axonal transport and Alzheimer’s disease. *Annu. Rev. Biochem.* 75 607–627. 16756504 10.1146/annurev.biochem.75.103004.142637

[B52] Sviatko K. Hangya B. (2017). Monitoring the right collection: the central cholinergic neurons as an instructive example. *Front. Neural Circ.* 11:31. 10.3389/fncir.2017.00031 28496401 PMC5406463

[B53] Thordardottir S. Graff C. (2018). Findings from the swedish study on familial Alzheimer’s disease including the APP Swedish double mutation. *J. Alzheimer’s Dis.* 64 S491–S496. 10.3233/JAD-179922 29614673

[B54] Tuszynski M. H. Thal L. Pay M. Salmon D. P. Hs U. Bakay R. (2005). A phase 1 clinical trial of nerve growth factor gene therapy for Alzheimer disease. *Nat. Med.* 11 551–555. 10.1038/nm1239 15852017

[B55] Tuszynski M. H. Yang J. H. Barba D. Hs U. Bakay R. A. Pay M. M. (2015). Nerve growth factor gene therapy: activation of neuronal responses in Alzheimer disease. *JAMA Neurol.* 72 1139–1147. 10.1001/jamaneurol.2015.1807 26302439 PMC4944824

[B56] Weissmiller A. M. Natera-Naranjo O. Reyna S. M. Pearn M. L. Zhao X. Nguyen P. (2015). A gamma-secretase inhibitor, but not a gamma-secretase modulator, induced defects in BDNF axonal trafficking and signaling: evidence for a role for APP. *PLoS One* 10:e0118379. 10.1371/journal.pone.0118379 25710492 PMC4339551

[B57] Xu W. Weissmiller A. M. White J. A. II Fang F. Wang X. Wu Y. (2016). Amyloid precursor protein-mediated endocytic pathway disruption induces axonal dysfunction and neurodegeneration. *J. Clin. Invest.* 126 1815–1833. 10.1172/JCI82409 27064279 PMC4855914

[B58] Zhang X. Bearer E. L. Boulat B. Hall F. S. Uhl G. R. Jacobs R. E. (2010). Altered neurocircuitry in the dopamine transporter knockout mouse brain. *PLoS One* 5:e11506. 10.1371/journal.pone.0011506 20634895 PMC2901340

