# Supplementary material for: Decoupling the Effects of the Amyloid Precursor Protein From Amyloid-β Plaques on Axonal Transport Dynamics in the Living Brain
Source: Front Cell Neurosci. 2019 Dec 3;13:501. doi: 10.3389/fncel.2019.00501 (PMC6901799; doi:10.3389/fncel.2019.00501)
Supplement: Supplementary file 1 [file Data_Sheet_1.pdf]

Decoupling the Effects of the Amyloid Precursor Protein from Amyloid-β Plaques on Axonal Transport Dynamics in the Living Brain

Medina, Uselman, Barto, Chaves, Jacobs and Bearer

Front. Cell. Neurosci. (2019) 13:501

Supplementary Materials

Supplementary Table S1: T-values for p<0.01 FDR SPM images shown in **Figure 6** and in the **Supplemental Video S1.**

Supplementary Table S2: Statistical significance of n-way ANOVA of between group, within group and main effects of time, condition and interaction across all region of interest measurements for each of the three locations shown in **Figure 7**.

Supplementary Fig. S1: Mouse tau protein is phosphorylated and aggregates in dystrophic neurites surrounding plaques in Group A and B.

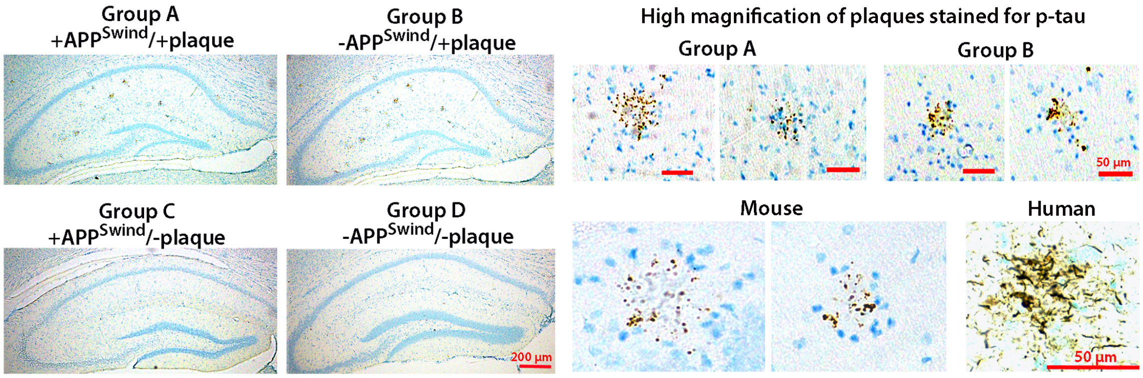

Figure S1. Mouse tau protein is phosphorylated and aggregates in dystrophic neurites surrounding plaques in Group A and B.

Phospho-tau staining with the AT8 monoclonal antibody detects dystrophic neurites in plaque-like aggregates in Group A and B but not in C or D (left panels). Higher magnification (right panels) shows p-tau deposition as aggregates of dot-like and linear structures in a 50-75 µm diameter cluster shown at 10x (top row) and 20x (bottom row). Control slides of advanced human Alzheimer's disease stained for AT8 in parallel show neurofibrillary tangles and more p-tau deposition throughout the brain as well as aggregates and neurites in plaques (lower right). Distribution of these clusters parallels that of Aβ plaques shown in **Figure 2**. These mice only carry the mouse tau gene. Hence mouse tau also becomes hyper-phosphorylated in the presence of Aβ plaques presumably composed of human Aβ derived from the APP^{Swind} transgene. The average number of clusters in the dorsal hippocampus of either Group A or B was 8.9 and 8 respectively, and no clusters were observed in Group C or D.

The fact that p-tau is found in Group B but not Group C is significant, since Group B

mice have better transport than those in Group C, which lack p-tau. This suggests, that at least for mouse, p-tau has minimal effect on transport and argues for other causes of transport defects. Thus our Group B mimics both spontaneous idiopathic Alzheimer's disease. In addition a recent report in a Familial Alzheimer's Disease due to the PSEN1 mutation demonstrates that it is possible to have a heavy burden of Aβ plaques and p-tau without dementia in a background of homozygous APOE3 with the Christchurch mutation (Arboleda-Velasquez et al., 2019). The Christchurch mutation interferes with APOE3 binding to heparan sulfate (and possibly other matrix proteins). We might speculate that differences in APOE allelic interactions with heparan sulfate affects transport, since heparan sulfate is transported by fast axonal transport ((Elam et al., 1970)) and this may be required for synaptic health (Elam and Ripellino, 1988).

Supplemental Method: The same brains imaged by MR were embedded en block (20 brains in a block, 5 for each cohort A-D, serially sectioned and alternate blocks stained as described in Methods and in (Bearer et al., 2018)). These are the same brains shown in **Figure 2** where they were stained by silver stain for plaques. In this case brains were stained for p-tau with the AT8 antibody, with an advanced human Alzheimer's disease specimen as a control by Neuroscience Associates. Images were captured on a Nikon microscope with DSL camera as described in Methods. Clusters were counted in three sections through the dorsal hippocampus in 5 animals for each Group.

Supplementary Video S1

Video shows a 3D rendering with segmented hippocampus and septal nuclei of injection site shown in **Figure 4** and the SPM results shown in **Figure 6**. The video begins with SPM overlay images captured at 30 m post-injection, which show the injection sites (projected as landmarks in Amira in **Figure 4**), continuing to the 6 h and 24 h images **(Figure 6**). Septal nuclei and hippocampus (grey mesh) are overlaid onto our template image (semi-transparent gray-scale brain). The four quadrants correspond to SPMs for each of the four groups; Group A (+APP^{Swind}/+plaques), top left, yellow; Group B (-APP^{Swind}/+plaques), top right, red; Group C (+APP^{Swind}/-plaques), bottom left, blue; Group D (wild-type, -APP^{Swind}/-plaques) bottom right, green ($p<0.01$, FDR corrected). Statistically significant voxels are highlighted, fading in and out for each successive time point beginning with the 30 m post injection, followed by 6 h, and lastly 24 h post-injection. For T-values please see **Supplemental Table 1**.

Arboleda-Velasquez, J.F., F. Lopera, M. O'Hare, S. Delgado-Tirado, C. Marino, N. Chmielewska, K.L. Saez-Torres, D. Amarnani, A.P. Schultz, R.A. Sperling, D. Leyton-Cifuentes, K. Chen, A. Baena, D. Aguillon, S. Rios-Romenets, M. Giraldo, E. Guzman-Velez, D.J. Norton, E. Pardilla-Delgado, A. Artola, J.S. Sanchez, J. Acosta-Uribe, M. Lalli, K.S. Kosik, M.J. Huentelman, H. Zetterberg, K. Blennow, R.A. Reiman, J. Luo, Y. Chen, P. Thiyyagura, Y. Su, G.R. Jun, M. Naymik, X. Gai, M. Bootwalla, J. Ji, L. Shen, J.B. Miller, L.A. Kim, P.N. Tariot, K.A. Johnson, E.M. Reiman, and Y.T. Quiroz. 2019. Resistance to autosomal dominant

Alzheimer's disease in an APOE3 Christchurch homozygote: a case report. *Nature medicine*.
Bearer, E.L., B.C. Manifold-Wheeler, C.S. Medina, A.G. Gonzales, F.L. Chaves, and R.E. Jacobs. 2018. Alterations of functional circuitry in aging brain and the impact of mutated APP expression. *Neurobiol Aging*. 70:276-290.
Elam, J.S., J.M. Goldberg, N.S. Radin, and B.W. Agranoff. 1970. Rapid axonal transport of sulfated mucopolysaccharide proteins. *Science (New York, N.Y.)*. 170:458-460.
Elam, J.S., and J.A. Ripellino. 1988. Association of axonally transported heparan sulfate with isolated synaptic plasma membrane. *Neurochemical research*. 13:715-720.
