# Supplementary material for: Decoupling the Effects of the Amyloid Precursor Protein From Amyloid-β Plaques on Axonal Transport Dynamics in the Living Brain
Source: Front Cell Neurosci. 2019 Dec 3;13:501. doi: 10.3389/fncel.2019.00501 (PMC6901799; doi:10.3389/fncel.2019.00501)
Supplement: Supplementary file 2 [file Table_1.pdf]

Supplementary Table S1.

T Values for Paired T Tests within SPM

Comparison	Group/Condition	FDR $p < 0.01$
**30 minutes > Pre-Injection**	Group A (+ APP^{SwInd} + Amyloid-β/plaques)	T = 6.49
	Group B (- APP^{SwInd} + Amyloid-β/plaques	T = 7.87
	Group C (+ APP^{SwInd} - Amyloid-β/plaques)	T = 5.00
	Group D (- APP^{SwInd} - Amyloid-β/plaques)	T = 6.12
**6 hours > 30 minutes**	Group A (+ APP^{SwInd} + Amyloid-β/plaques)	T = 5.76
	Group B (- APP^{SwInd} + Amyloid-β/plaques)	T = 8.34
	Group C (+ APP^{SwInd} - Amyloid-β/plaques)	T = 5.75
	Group D (- APP^{SwInd} - Amyloid-β/plaques)	T = 5.41
**24 hours > 30 minutes**	Group A (+ APP^{SwInd} + Amyloid-β/plaques)	T = 4.90
	Group B (- APP^{SwInd} + Amyloid-β/plaques)	T = 6.12
	Group C (+ APP^{SwInd} - Amyloid-β/plaques)	T = 4.98
	Group D (- APP^{SwInd} - Amyloid-β/plaques)	T = 4.60
