# Supplementary material for: Decoupling the Effects of the Amyloid Precursor Protein From Amyloid-β Plaques on Axonal Transport Dynamics in the Living Brain
Source: Front Cell Neurosci. 2019 Dec 3;13:501. doi: 10.3389/fncel.2019.00501 (PMC6901799; doi:10.3389/fncel.2019.00501)
Supplement: Supplementary file 3 [file Table_2.DOCX]

| **Supplemental Table 2.** | | | | | | | | | | | | | | |
| --- | --- | --- | --- | --- | --- | --- | --- | --- | --- | --- | --- | --- | --- | --- |
| **Two Way ANOVA** | | | | | | | | | | | | | | |
| **Main Effect** | **Fimbria** | | | | | **Lateral Septum** | | | | **Orbital Prefrontal Cortex** | | | | |
|  | **F(DFn, DFd)** | **P value** | | | **Significance Level** | **F(DFn, DFd)** | **P value** | | **Significance Level** | **F(DFn, DFd)** | **P value** | | | **Significance Level** |
| **Interaction** | F_(6,80)_ = 5.328 | 0.0001 | | | *** | F_(6,80)_ = 4.135 | 0.0011 | | ** | F_(6,80)_ = 3.381 | 0.0051 | | | ** |
| **Time** | F_(2,80)_ = 81.19 | < 0.0001 | | | **** | F_(2,80)_ = 50.81 | < 0.0001 | | **** | F_(2,80)_ = 42.16 | < 0.0001 | | | **** |
| **Condition** | F_(3, 40)_ = 7.525 | 0.0004 | | | *** | F_(3,40)_ = 4.546 | 0.0078 | | ** | F_(3,40)_ = 4.837 | 0.0058 | | | ** |
| **Between Group - Multiple Comparisons - Bonferroni Corrected** | | | | | | | | | | | | | | |
| **30m** | **P value** | | **Significance Level** | | | **P value** | | **Significance Level** | | **P value** | | **Significance Level** | | |
| **A vs B** | > 0.9999 | | ns | | | > 0.9999 | | ns | | > 0.9999 | | ns | | |
| **A vs C** | > 0.9999 | | ns | | | > 0.9999 | | ns | | > 0.9999 | | ns | | |
| **A vs D** | > 0.9999 | | ns | | | > 0.9999 | | ns | | > 0.9999 | | ns | | |
| **B vs C** | > 0.9999 | | ns | | | > 0.9999 | | ns | | > 0.9999 | | ns | | |
| **B vs D** | > 0.9999 | | ns | | | > 0.9999 | | ns | | > 0.9999 | | ns | | |
| **C vs D** | > 0.9999 | | ns | | | > 0.9999 | | ns | | > 0.9999 | | ns | | |
| **6h** | **P value** | | | **Significance Level** | | **P value** | | **Significance Level** | | **P value** | | | **Significance Level** | |
| **A vs B** | 0.1895 | | ns | | | 0.0049 | | ** | | > 0.9999 | | ns | | |
| **A vs C** | 0.1059 | | ns | | | > 0.9999 | | ns | | > 0.9999 | | ns | | |
| **A vs D** | 0.0309 | | * | | | > 0.9999 | | ns | | 0.0044 | | ** | | |
| **B vs C** | 0.0001 | | *** | | | 0.0009 | | *** | | 0.0696 | | ns | | |
| **B vs D** | < 0.0001 | | **** | | | 0.0003 | | *** | | 0.4851 | | ns | | |
| **C vs D** | > 0.9999 | | ns | | | > 0.9999 | | ns | | < 0.0001 | | **** | | |
| **6h** | **P value** | | | **Significance Level** | | **P value** | | **Significance Level** | | **P value** | | | **Significance Level** | |
| **A vs B** | > 0.9999 | | ns | | | > 0.9999 | | ns | | > 0.9999 | | ns | | |
| **A vs C** | 0.0013 | | ** | | | 0.0344 | | * | | > 0.9999 | | ns | | |
| **A vs D** | 0.2150 | | ns | | | 0.6498 | | ns | | 0.0221 | | * | | |
| **B vs C** | < 0.0001 | | **** | | | 0.0009 | | *** | | > 0.9999 | | ns | | |
| **B vs D** | 0.0068 | | ** | | | 0.0340 | | * | | > 0.9999 | | ns | | |
| **C vs D** | 0.5507 | | ns | | | > 0.9999 | | ns | | 0.0319 | | * | | |
| **Within Group - Multiple Comparisons - Bonferroni Corrected** | | | | | | | | | | | | | | |
| **Group A** | **P value** | | | **Significance Level** | | **P value** | | **Significance Level** | | **P value** | | | **Significance Level** | |
| **6h vs 30m** | < 0.0001 | | | **** | | 0.0009 | | *** | | 0.0081 | | | ** | |
| **24h vs 30m** | < 0.0001 | | | **** | | < 0.0001 | | **** | | 0.0452 | | | * | |
| **24h vs 6h** | 0.0407 | | | * | | 0.4976 | | ns | | > 0.9999 | | | ns | |
| **Group B** | **P value** | | | **Significance Level** | | **P value** | | **Significance Level** | | **P value** | | | **Significance Level** | |
| **6h vs 30m** | < 0.0001 | | | **** | | < 0.0001 | | **** | | 0.0002 | | | *** | |
| **24h vs 30m** | < 0.0001 | | | **** | | < 0.0001 | | **** | | 0.0018 | | | ** | |
| **24h vs 6h** | 0.0088 | | | ** | | 0.6113 | | ns | | > 0.9999 | | | ns | |
| **Group C** | **P value** | | | **Significance Level** | | **P value** | | **Significance Level** | | **P value** | | | **Significance Level** | |
| **6h vs 30m** | < 0.0001 | | | **** | | 0.0017 | | ** | | 0.2212 | | | ns | |
| **24h vs 30m** | > 0.9999 | | | ns | | 0.1671 | | ns | | 0.0106 | | | * | |
| **24h vs 6h** | < 0.0001 | | | **** | | 0.3068 | | ns | | 0.7079 | | | ns | |
| **Group D** | **P value** | | | **Significance Level** | | **P value** | | **Significance Level** | | **P value** | | | **Significance Level** | |
| **6h vs 30m** | 0.0003 | | | *** | | 0.0147 | | * | | < 0.0001 | | | **** | |
| **24h vs 30m** | 0.0642 | | | ns | | 0.0039 | | ** | | < 0.0001 | | | **** | |
| **24h vs 6h** | 0.2624 | | | ns | | > 0.9999 | | ns | | 0.6334 | | | ns | |
